# Complete Genome Sequences of Two Enterococcus faecalis Bacteriophages, EFGrKN and EFGrNG, Targeted to Phage Therapy

**DOI:** 10.1128/MRA.00126-21

**Published:** 2021-04-22

**Authors:** Sivan Alkalay-Oren, Naama Gold, Leron Khalifa, Ortal Yerushalmy, Shunit Coppenhagen-Glazer, Ran Nir-Paz, Ronen Hazan

**Affiliations:** a Institute of Dental Sciences, Hebrew University of Jerusalem, Jerusalem, Israel; b Department of Clinical Microbiology and Infectious Diseases, Hadassah-Hebrew University Medical Center, Jerusalem, Israel; Loyola University Chicago

## Abstract

EFGrKN and EFGrNG are new Enterococcus faecalis phages that were isolated from sewage samples as part of the Israeli Phage Bank (IPB). The complete genomes were sequenced, analyzed, and deposited in GenBank. According to their lytic activity *in vitro*, it seems that these phages have a potential to be used in future phage therapy treatments.

## ANNOUNCEMENT

The genus *Enterococcus* includes 38 species of Gram-positive bacteria, among which E. faecalis and E. faecium are the most abundant pathogens in humans. They are usually part of the human intestine and female genital tract microbiota and are considered to be commensals (1). Both species can also cause many infections, including urinary tract infections, endocarditis, bacteremia, wound infections, meningitis, and more (2). These two *Enterococcus* species are ranked among the most common and resistant pathogens in hospitals (3). As such, enterococci, mainly vancomycin-resistant strains, are an appropriate target for phage therapy (4).

**FIG 1:**
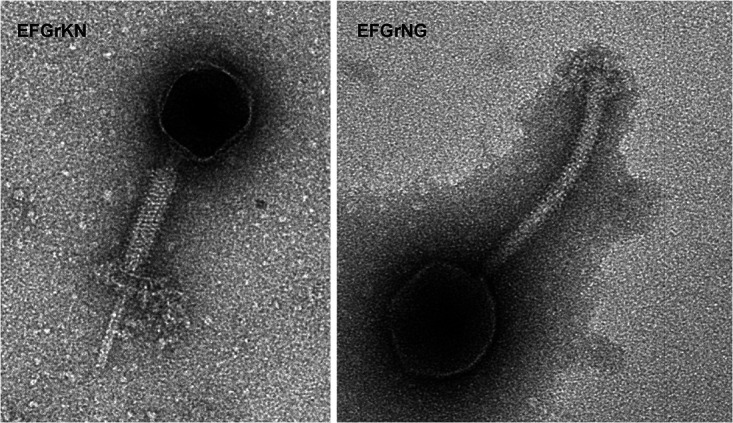
TEM visualization of the phages EFGrKN and EFGrNG.

Here, we report the isolation of two new E. faecalis phages, EFGrKN and EFGrNG. Both were isolated from a 5-liter sewage sample obtained from the Sorek West Jerusalem sewage decontamination facility located in central Israel. Purification was conducted using the phage titration method, as previously described (5, 6). Briefly, the sewage samples were centrifuged at 7,100 × *g* for 10 min, and then the supernatants were filtered using a 0.22-μm microfilter (Sartorius). Then, 500 µl of overnight-grown E. faecalis V583 in brain heart infusion (BHI) medium was mixed with 10 ml of BHI medium and 10 ml of filtrate. The mixture was incubated at 37°C for 24 h. The resulting cultures were centrifuged at 7,100 × *g* for 10 min, and the supernatant was filtered using a 0.22-μm microfilter. A plaque assay was used to identify phages (5); 10 µl of the enriched filtrate was spotted on bacterial lawns of E. faecalis V583, and after 24 h of incubation at 37°C, clear plaques were observed. In order to obtain a pure phage, 3 rounds of isolation seeds were performed on the same strain. The phages were visualized using transmission electron microscopy (TEM) (Fig. 1) as described previously (5).

DNA was purified using a phage DNA isolation kit (Norgen Biotek), and its concentration was measured with a Qubit fluorometer using a Qubit double-stranded DNA (dsDNA) high-sensitivity assay (both from Invitrogen, Carlsbad, CA, USA). A concentration of 1 to 30 ng total DNA was used for DNA sequencing library preparation. The sequencing of EFGrNG was performed using Oxford Nanopore MinION and Illumina technologies. The sequencing of EFGrKN was performed only with Illumina technology. For the MinION sequencing, libraries were prepared using the 1D native barcoding genomic DNA (EXP-NBD104, EXP-NBD114, SQK-LSK109), and samples were sequenced using an R9.4.1 flow cell (FLO-MIN106). Demultiplexing was performed using EPI2ME. The sequencing of EFGrNG yielded 173,851 long reads ranging from 24 to 63,432 bp.

For the Illumina method, libraries were prepared using the Nextera XT kit (Illumina) with DNA fragmented, tagged, cleaned, and normalized according to the manufacturer’s recommendations. Library purity and quantity were evaluated with a 2200 TapeStation system (Agilent Technologies, Inc., Santa Clara, CA, USA) using a D1000 ScreenTape kit (Agilent Technologies, Inc.) and with a Qubit fluorometer (Invitrogen, Carlsbad, CA, USA) using a Qubit dsDNA high-sensitivity assay (Invitrogen). The DNA was diluted to a concentration of 4 nM. Reads of more than 100× depth for each sample were targeted. Samples were deep sequenced on a NextSeq 500/550 machine as single-end reads of 150 bp using the 150-cycle midoutput kit v2 (Illumina, San Diego, CA, USA). Binary base call (BCL) files as machine output were converted to FASTQ files using the bcl2fastq/2.20.0 program.

Most bioinformatic analysis was performed using Geneious Prime 2021.0.3 build 2020-12-23 and its plugins (Biomatters). The reads were trimmed using the Geneious Prime BBDuk adapter/quality trimming program v38.84 by Brian Bushnell with default parameters. The sequencing of EFGrNG and EFGrKN yielded 12,625,612 and 13,331,382 reads, respectively, of 150-bp size.

The MinION reads of EFGrNG were first *de novo* assembled using the Flye plugin of Geneious Prime 2020 v1.1 (https://github.com/fenderglass/Flye) in order to create a backbone genome sequence. Then, the Illumina reads were mapped to the backbone sequence using the Geneious assembler. For EFGrNG, *de novo* assembly was performed on 20% of random reads with the SPAdes v3.13.0 plugin of Geneious Prime with default parameters. The resulting contigs were reassembled with the Geneious Prime assembler with the highest sensitivity.

Both draft genome sequences were fine-tuned by mapping to the reference of all the Illumina reads, which resulted in average coverage of 5,025× (EFGrNG) and 3,806× (EFGrKN).

The genomes of EFGrKN and EFGrNG were both found to be circular and contained 147,532 bp (37.3% GC content) and 145,199 bp (37% GC content), respectively. EFGrKN has 190 coding DNA sequences (CDS) and 25 tRNA genes, and EFGrNG has 105 CDS and 22 tRNA genes. Annotation using RAST (7) revealed that most of these genes code for phage-related or hypothetical proteins. Alignment of the two phages with BLAST (https://blast.ncbi.nlm.nih.gov/Blast.cgi) shows that the phages’ coverage is 95% with 97.8% identity. In addition to several point mutations, there are some deletions/insertions of larger regions; most of those variations are in hypothetical proteins or uncharacterized phage proteins. Interestingly, there are two recognizable differences; tRNA-Val was found only in EFGrKN, and the gene for deoxyadenosine kinase has a large deletion in EFGrNG. The phages are phylogenetically classified as Duplodnaviria, Heunggongvirae, Uroviricota, Caudoviricetes, Caudovirales, Herelleviridae, Brockvirinae, and Kochikohdavirus and display high similarity to other phages from this subfamily, including EFDG1 (GenBank accession number NC_029009.1), which we isolated previously (5).

As potential candidates for phage therapy treatments, the phages were scanned for virulence factors with ABRicate v1.0.1 (https://github.com/tseemann/abricate) using all its databases with its default parameters. No virulence factors or resistance genes were identified, and therefore it seems that these phages are safe for use in future phage therapy treatments.

### Data availability.

These two genome sequences were deposited in GenBank; EFGrKN’s accession number is MW004544.1, and EFGrNG’s accession number is MW004545.1. The raw data were deposited in the NIH BioSample database project (BioProject number PRJNA706131)/Israeli Phage Bank (IPB), with accession numbers SAMN18137778 for EFGrKN and SAMN18191644 for EFGrNG.
